# Australian Public Opinions Regarding the Live Export Trade before and after an Animal Welfare Media Exposé

**DOI:** 10.3390/ani8070106

**Published:** 2018-06-29

**Authors:** Michelle Sinclair, Tessa Derkley, Claire Fryer, Clive J. C. Phillips

**Affiliations:** Centre for Animal Welfare and Ethics, School of Veterinary Science, University of Queensland, Gatton, QLD 4343, Australia; tessa.derkley@uq.net.au (T.D.); claire.fryer@uq.edu.au (C.F.); c.phillips@uq.edu.au (C.J.C.P.)

**Keywords:** live export, animal welfare, public opinion, exposé, cruelty footage

## Abstract

**Simple Summary:**

Long distance transport of livestock from one continent to another by ship raises concerns about the welfare of the animals on board the ship. Media exposés may have influenced the public towards negative views about the trade. A total of 522 members of the public in Brisbane, Australia, were surveyed just before and after an exposé of cruelty to sheep on board ships destined for the Middle East in 2017. More respondents had negative than positive attitudes towards the trade and almost one half had seen the media exposé. The exposé increased the proportion of respondents indicating that they were familiar with the trade, and although it did not affect those indicating negative feelings towards it, it increased the proportion believing the trade should end.

**Abstract:**

The long distance export of livestock from Australia to Asia has long aroused controversy for suspected animal welfare concerns during and after the voyage. However, there is little or no information on the attitude of the Australian public towards this trade. A total of 522 Australians were surveyed in Brisbane to find out about their understanding of the trade, their attitudes towards it and the influence of demographic factors. Approximately one half of respondents were surveyed just before a media exposé of cruelty on sheep shipments in 2017 from Australia to the Middle East and one half just after the exposé, to see the impact of media depiction of cruel treatment of live export sheep. Most respondents believed that they were familiar with the industry, and more after the media exposé than before. More respondents had negative than positive feelings about the trade, and just over a quarter had no feelings. Twice as many thought it should be ended than maintained, particularly women, but 40% said that it depends, mainly on ethics and animal-based reasons. Those that thought it should not be ended mainly did so to support farmers and the country’s economy. Almost one half had seen the media exposé, particularly older respondents, and expressions of sadness, empathy for the animals and anger were the most common responses to such footage. Although it increased the number of people saying that they were familiar with the trade, it did not affect people’s view of the trade, except that fewer indicated that ending the trade was dependent on other factors. It is concluded that the majority of Australian respondents in one capital city had negative views towards the live export trade, and that a media exposé had some influence on this view.

## 1. Introduction

Over 2.6 million animals are shipped annually alive from Australia to more than 60 countries, such as Indonesia, Vietnam and the Middle East [1] in a trade that has been running for over 150 years and is currently worth over Aus $800 M per annum [2]. The Australian live export industry, however, has been the focus of public scrutiny and systematic government reviews and policy debate since 1985 [3]. The primary debate revolves around the primary stakeholders at the centre of the trade; the animals and their welfare. Through science, standards of animal welfare can be assessed objectively. Findings show that long ship journeys are stressful for cattle and sheep, who are accustomed to an existence on land, and, in many cases, it can be established that they may experience suffering to some degree for the entire journey of up to three weeks duration [4]. There are many factors that comprise this suffering—and most are attributable to conditions on the ship. This can include ammonia build up from urination in highly stocked closed spaces [4], which causes mucosal irritation and pulmonary inflammation [4]; the high stocking density predisposing animals to heat stress [4,5]; inability to access food and water as required [6,7]; and an inability to lay down and rest [6]. In the unlikely case that all of these conditions were able to be mitigated for improved welfare, the more recently discovered issue of seasickness provides grounds for further concern over poor welfare [8]. Other conditions attributable to poor stockmanship may potentially occur at any stage of the animal’s journey and carry serious concerns regarding poor animal welfare, such as a failure to identify pregnant females before embarkation, who subsequently give birth to lambs on the ships, which may then be thrown overboard. Animal welfare concerns also arise when the animals reach their destination, at which point they may be subject to methods of handling, further transportation and ultimately slaughter that are not consistent with Australian standards, and which cause high levels of stress, pain and suffering [9]. The conditions the animals encounter when subjected to live export was not a matter debated in the political sphere until a series of accidents on livestock carriers in the 1980s [3]. The first review stated “... if a decision were to be made on the future of the trade purely on animal welfare grounds, there is enough evidence to stop the trade” [10]. Since 1985, Australian live trade export has continued to be heavily debated at key times. In 2003, the trade to Saudi Arabia was ceased for two years in response to a disease outbreak affecting Australian animals in the country [11], and again in 2006, in response to footage showing cruel treatment of Australian animals in an Egyptian slaughterhouse, trade to the country was suspended [12]. In 2011, a televised “Four Corners” exposé of cruel treatment of Australian animals in Indonesian slaughterhouses resulted in a six-week suspension to the entire trade [3]. In 2018, the Australian Agriculture Minister responded to a televised exposé on the conditions and deaths of over 2400 sheep on a shipment to the Middle East (8 May 2018) by stating “Even if the circumstances can be explained, these deaths are plainly unacceptable,” [13].

In addition to the animals, the stakeholders of the live export trade include agricultural enterprises, particularly in Northern Australia. “[t]his trade is highly significant to rural Australia. It stabilises the value of all sheep by providing competition through additional markets thus helping to cushion the effects of droughts and broadening the range of management strategies available to the sheep industry for both livestock and crops” [10]. Another industry body states that “over 75% of properties (in northern Australia) reported to be partially or completely reliant on live cattle receipts” [14]. At each of these critical times of live export debate, these economic considerations have weighed against animal welfare considerations and have resulted in an end to trade suspensions, albeit with some reforms, such as the introduction of the Exporter Supply Chain Assurance System (ESCAS). “The Committee agreed that the animal welfare aspects of the trade cannot be divorced from economic and other considerations… After consideration of all factors, the Committee acknowledges the reality of the situation that any short-term cessation or disruption to the trade would cause considerable dislocation both in Australia and in the Middle East” [10]. However, substantial questions exist around the effectiveness of ESCAS as a safeguard for animal welfare [15], and challenges to the economic arguments now exist that suggest the Australian economy will not lose money in the long term with a switch to a carcase trade (slaughtering the animals in Australia and sending meat rather than live animals).

In South Australia, which is Australia’s second biggest live sheep exporting state, the Australian Meat Industry Employees Union claims that, if all sheep were slaughtered in the state’s abattoirs, the chilled meat trade would be worth about $500 million, with their representative recently stating “We have the capacity, we just need leadership from the government to make it happen” [16]. However, this is contested, and, while an end to the live trade may offer opportunities for new business, jobs and growth in Australian meat processing, it would be permanently detrimental to enterprises specialising in live export. Furthermore, if the live trade did cease instantaneously, the potential exists for such a change to be temporarily detrimental to farmers who have focused their business on raising animals specifically for live export [17].

There is little doubt that many of the arguments around live export are polarised and fiercely (and often emotionally) defended from both the animal welfare lobby and the live export industry, leaving the Australian Government in a position to consider both interests alongside science and economic review. While the positions of lobbyists on each side are clear, what has thus far been missing are the opinions of the Australian general public; those who democratic governments are elected to represent. Although polls by news media and NFPs have been conducted, an independent and balanced survey of the general public has previously been unavailable. A study has surveyed members of the public who had encountered media broadcasts of animal cruelty of cattle exported for slaughter [18], however this did not take into account the opinions of those who had not seen this footage—therefore, it was not an overall reflection of public views. In addition to this, the study only offered respondents the opportunity to agree or disagree with negative emotional responses to the footage, and the sample size of 157 was small.

This study aimed to provide independent academic information on public opinion about live export, as assessed both immediately before and after the wide media release of the exposé of cruelty to sheep sent from Australia to the Middle East in 2018. This will support public debate regarding the Australian live export trade, and will be of assistance to policy makers tasked with addressing the issue.

## 2. Materials and Methods

This study was approved by the University of Queensland Ethics Committee (approval number 2018000278).

### 2.1. Questionnaire Delivery

Respondents were selected at random during two street survey days. The first date, 7 April 2018, was selected to occur prior to release of new footage from a live export ship. The second date, 14 April 2018, was selected as the weekend following the release of this footage.

Participants were approached at random across a number of Brisbane CBD locations to allow for a diverse respondent base. Each surveyor first asked the potential participant/s if they had a spare 2 min to complete an independent survey through the University of Queensland to gather their opinion on the live-export industry in Australia. If the request was accepted, the surveyor then asked if the respondents classed themselves as Australian. As this study is attempting to reveal and present the Australian public opinion on the live export trade, if they did not personally identify as Australian, they were thanked for their time and no further study was undertaken. Therefore, only Australians were included in this survey.

### 2.2. Questionnaire Creation and Design

The survey was created using the online software tool, Survey Monkey, and was designed to allow anonymous participation. The survey comprised 13 questions in total. Question 1 asked the respondent if they were familiar with the live-export industry in Australia, and if they answered negatively they were told “live export is the trade in which we send live Australian animals, usually by ship, to other countries”. Question 2 asked how they felt about this industry, with the response options positive, negative and no feelings. Following this, Question 3 asked if they had seen recent footage pertinent to the live-export debate; if they answered “yes” then they were taken to Question 4—a multiple-choice question that asked each relevant respondent what their response to the footage was. If they answered “no” to Question 3, they were directed to Question 5. Question 5 asked if the respondent believed the live-trade industry should be ended—“yes”, “no”, or “depends” were the response options. If Question 5 was answered with “yes”, they were directed to Question 6 where they were asked if they believed the live-trade industry should be phased out or if it should end instantly. If “no” was the answer to Question 5, then the respondent was taken to Question 7, a multiple choice question asking for their single main reason for saying the live-trade industry should not be ended. If the respondent selected “depends” in Question 5, then they were taken to Question 8, a multiple choice question to establish the main reason for selecting “depends” to whether the live-trade industry should be ended. Questions 6–8 were all multiple choice questions. Questions 9–13 were demographic questions, which asked the respondents their gender, their age group, if they worked within the livestock industry, their residential zone, and their highest education level, respectively. Depending on the respondent’s answer to each question, each individual answered either 10 or 11 questions. The survey was designed in such a way that it should have taken, on average, approximately two minutes to complete.

## 3. Statistical Analysis

Data were entered into an Excel spreadsheet and analysed in Minitab. It was first screened for completeness and possible false answers. No respondents’ answers had to be rejected due to incomplete data or lack of co-operation with the researchers. The number of survey responses in each response category before and after the media event were compared by the Chi square test. Survey responses were analysed for the significance of demographic effects using binary, ordinal and nominal logistic regression models of output variables as appropriate. The regression models employed the log likelihood method. Age was entered as an ordinal variable, education level and residential zone as categorical, and gender and working in the industry as binary.

## 4. Results

A total of 522 respondents participated in the study, 280 on Day 1, and 242 on Day 2. A description of general public respondent demographics (Table 1) shows that 59% were female, 51% lived in suburbs, 32% had a degree or were studying towards one, and only 3% worked in the livestock sector. In comparing respondent demographics to Australians as a whole, 49.3% of Australians are male, compared with 41% respondents, and 50.7% Australians are female, compared with 59% respondents [19]. The median age of Australians is 38 [19], with the most common age range of respondents 30–39. Fourteen per cent of respondents lived rurally, with approximately 32% of Australians in general living rurally [19]. Overall, 16.1% of Australians have attended university or tertiary studies [19] compared with 55% of respondents stating attendance (including post graduate).

A total of 74% stated they were familiar with the live export trade, 25% were not. There was a significant association (*p* = 0.05) between the timing of the survey (before or after the media event) and the proportion stating that they were familiar with the industry, with the latter increasing after the event (Table 2). Sixty per cent of respondents felt negative about the trade, 14% positive and 26% had no feelings, proportions that were not associated with timing of the survey (*p* = 0.16). Overall, 48% said that they had seen the recent footage around the live export debate, 53% had not. The number of respondents saying that they had seen footage associated with the live export trade increased from 42% to 54% in the survey after the exposé event (*p* = 0.006) that is subject of this study. Forty-two per cent believed the trade should be ended, 21% that it should not, with 40% saying that it depends on something. Of those that said the trade should be ended or that ending it was dependent on some factor, 51% said it should be ended instantly, 49% that it should be phased out.

Neither response (ending the trade and instant/phase out) was dependent on when the survey was conducted. However, those that had seen the footage were more likely to indicate that live export should be ended and less likely to state that it depends (Table 3).

When those who had seen the recent footage were asked how they felt about it, their single greatest response was a feeling of “sadness” (22.5%) (Table 4). Nearly 20% said that they mostly felt empathy towards the animals, 15% felt “anger”, and 13% said that it was “sickening”. Much fewer people indicated that they were in disbelief, frustrated, disgusted, disappointed, irritated at the sensationalism, in shock, sick of hearing about it or defensive of the industry.

Of those who answered “no”, or “depends” when asked if the trade should end (Table 5), the primary reasons cited were “farmer support” (45%) or “economy” (40%) for the “no” respondents, and for “depends”, respondents stated ending the trade depends on “ethical reasons” (34.5%), animal based reasons (38%) and to a lesser extent, “farmer support” (17.5%).

When analysing responses by demographic indicators (Table 6), both age and education level were statically significant (*p* < 0.05) in response to familiarity with the live trade industry.

Respondents 40 and over were more likely to be familiar with the industry than those under 40 (Table 7). Those with tertiary education were more likely to say that they were familiar than those educated to High School level only (per cent saying yes they were familiar was High School 64%, Technical College 74%, Bachelor’s degree 77%, postgraduate degree 82%). Older respondents were more likely to have seen the recent footage and more likely to have positive feelings towards the industry and less likely to have negative or no feelings. Respondents under 40 were more likely not to have seen the recent footage. Females were more likely to say that the live export industry should be ended (*n* = 150, 49% of females; males *n* = 69, 32% of males), than not ended (females *n* = 124, 40% of females; males *n* = 83, 39% of males), and they were less likely to say that it depends (females *n* = 34, 11% of females; males *n* = 61, 29% of males).

## 5. Discussion

The primary finding from this independent study is that the largest proportion of the Australian public have negative opinions towards the live export trade. Further to that, this study indicates that many more Australians want the trade to end than those who wish it to continue. This sentiment is most likely born from the systematic public and political debate around animal welfare, further supported by the importance of “animal-based reasons” as given by respondents in this study. This finding echoes polls conducted throughout the debate—with a recent RSPCA poll finding almost 73% of Australians wanting the trade to end altogether [20] and a news poll in The Western Star resulting in 82% supporting a ban on live export [21]—however, to a less polarised extent. As the Western Star is a rural publication, this suggests that rural dwellers are just as concerned over these revelations as city dwellers. This was also indicated in the survey results, with no significant difference between those in other residential zones when responding to the question of whether the trade should be banned.

This study shows a larger than expected number of Australians being unsure about the industry, or exhibiting a lack of knowledge around the industry, with a quarter of respondents stating they “had no feelings” about the live export trade.Interestingly, the same quantity of respondents (25%) stated they were not familiar with live export, suggesting a requirement for more information and education on the issue.

In support of this, researchers noted during data collection that many respondents stated that they felt they did not know enough about the trade to make a fully informed view, and hence may have been more inclined to answer with “no feelings”. Researchers also noted that many respondents were not familiar with the term “live export”, however showed more awareness when provided the brief definition given in the Methods. This may suggest a need to reassess the use of the term “live export” without definition, and ensure information surrounding the debate is simplified and accessible to the public.

Significantly, a large portion responded to the question of ending live export with “depends”, many of whom had reported negative feelings about the industry earlier in the survey. This may indicate a deeper understanding that the issue may not be a simple one, and potentially a condemnation of extreme opinions. The opinion “depends” was mostly given for animal-based reasons, with researchers reporting accompanying verbal responses such as, “depends if the animals can be provided for so that they don’t suffer at all”. As stated, the scientific literature demonstrates that, when assessing the trade from an animal based viewpoint, it is considered unlikely that the animals can be subjected to an economically viable live export without a level of suffering. Those that responded that the industry should not be ended referred to “availability of jobs”. With an end to the trade and a transition to local processing, it can be argued that more jobs would become available in Australia, rather than fewer. This gap may indicate a need for more exposure to these facts and more information, and suggests that the debate should be supported by clear and balanced scientific (animal-based) and economic (industry-based) facts.

This study also found that seeing footage was statistically associated with a belief that trade should be banned, however many had not watched footage. While this could be due to a lack of awareness that the footage exists, it also seems likely that a large proportion of these respondents knew of the footage and simply choose not to expose themselves to it in order to avoid the negative feelings associated with viewing the material, such as “sadness”, “sickness” and “anger” along with “empathy for the animals”. Researchers noted that verbal responses to this question included comments such as “No, can’t bear to watch it”. This suggests that, along with a need for increased knowledge around the live export industry, media other than graphic footage could encourage a wider audience and reduce the cognitive dissonance that may be occurring. This could potentially include the use of still images, more delivery of information via non-visual communication channels, such as radio debates, further information on the cognitive ability of transported animals and their ability to experience suffering, rather than a focus on the visual aspects of the suffering, and the use of comparative statistics to show how much relative space/food, etc., a sheep requires to be comfortable. This current survey also shows that young people were less likely to view the exposé but more likely to have negative responses to the trade. Finding other media than television to inform young people is clearly necessary.

It is likely that the public may respond differently to information, depending on how it is presented. For example, if the figure of total sheep deaths on live export ships in 2017 is presented simply as 0.71% [1], it may inspire a more positive response towards the industry, while, if presented as the death of 12,377 sheep [1], followed by the story of just one such animal, it may provoke a more negative response. With regards to human–human empathy, it has been suggested that people “often become numbly indifferent to the plight of individuals who are ‘one of many’ in a much greater problem” [22], therefore focusing on an individual animal’s suffering causes a more significant emotional response, rather than focusing on the suffering of the animal lost in the herd [23].

This study shows that the media release of video footage results in emotional responses and an increase in negative emotion, but may not result in support to end trade. A recent poll in the Stock Journal publication found that almost two thirds of respondents believed that the recent “60 Minutes” report was likely to threaten the future of the live sheep trade [24], which indicates concern within the industry for the effects of such footage. This was highlighted in a recent opinion piece by the farming publication The Weekly Times, which challenged claims that Australia’s live animal exporters are the “best in the world”, and care more for the animals than others may, and suggested that it is time to scrutinise claims from both sides of this debate [25].

The high proportion of respondents reporting “sadness” and “empathy for the animals” is not surprising. One author summarised the Australian attitude to animals by stating “in both the general community and amongst professionals working with animals, there is a majority view that animal welfare is a significant issue but that use of animals is acceptable provided that it does not lead to unnecessary suffering” [26].

While Australians clearly care about animals, they also care about the farmers involved in the trade. Whilst there is little doubt that the scientific evidence around animal experiences during the live export trade (separate to the suffering appearing in media exposés) supports the view that suffering does occur on the shipments, the purported economic outcomes surrounding a hypothetical cessation are less clear. Some evidence is available which suggests that the Australian economy would not be damaged in the long term with an industry switch to a trade in carcasses [17]. Some bodies advocate an end to the trade in order to increase economic benefit domestically through increased jobs in domestic meat processing. Meat processors in Western Australia (Australia’s biggest live sheep exporter) argue that the additional meat, which could then be processed onshore, could “be absorbed by lucrative and expanding export markets, such as the United States and those in the Middle East and Asia” [27]. This may be true if the export of lamb and mutton products are constrained by the number of sheep available for processing [27]. Meanwhile, an ACIL Tasman report into the live cattle trade from Northern Australia commissioned in 2012 by World Animal Protection (then WSPA) found that there would be a number of economic benefits for both producers and the Australian economy if more animals were processed domestically [28]. However, others forecast a cessation in trade to be economically damaging, with economic modelling and analysis conducted by the Centre for International Economics (CIE) for Meat and Livestock Australia and Livecorp finding that Australian livestock exports generate around 10,000 jobs across Australia and that “a cessation of the trade would impose a net cost of about $300 million annually on Australian livestock producers” [17]. The Australian Farm Institute does, however, acknowledge that “a limitation of this research is that it did not examine some of the flow-on or multiplier effects of a cessation of the trade. These would arise both in regions negatively impacted by the cessation of the trade (a negative impact), and in regions positively impacted by the resultant increase in meat processor throughput (a positive economic impact)” [17]. As “economy”, “farmer support” and “jobs” were the primary reasons cited amongst those in support of keeping the trade, rigorous and balanced information regarding financial values, repercussions and gains should be made available to the general public.

## 6. Limitations to the Study

While the sample size is substantially larger than the previously comparable study utilising the same methodology, the study surveyed a relatively small sector of the population within one of the major Australian cities. Whilst most Australians live in five major cities, the sampling was conducted over the weekend to capture some Australians visiting the city, and the sample group was diverse in terms of residential zone (suburbs, rural and city); however, we cannot be sure that the participants surveyed in Brisbane are typical of all Australians. It also cannot be certain that those who agreed to partake in the study when stopped at random did not have a particular interest in the live export; however, the results suggest this may not be the case. In addition, the individual respondents surveyed before and after the media event do not remain identical, therefore a general sentiment is reported rather than individual change of opinion. A larger sample size and re-survey of the same respondents may have been possible through a survey by telephone or email, however, due to the reducing usage of landline phones, the lack of publicly-listed mobile phone number databases, the poor response rate to non-personal telephone enquiries in Australia, a general unwillingness to divulge personal numbers to non-personal entities for follow up calls, and the potential biases present in online “snowballing” surveys, this study opted for a randomised in-person public approach on both days. Finally, a difference between attitudes to live export between rural and urban respondents is empirically echoed in some studies investigating attitudes to animal welfare (although not consistently). Rural residents may have a greater interest in this study due to proximity of farming activities tied to the trade.

## 7. Conclusions

The Australian public exhibited empathy towards animals in live export, and for this reason many supported an end to live export. However, given support for the local agriculture industry, a phase out is likely to be supported rather than an instantaneous cessation. Unlike previous media and advocacy opinion polls, this independent and scientific study shows that public opinion on this issue is divided. While a majority expressed negative feelings towards the industry, and the largest proportion indicated an interest in seeing the trade ended, many Australians indicated that they “had no feelings” on the matter, which probably reflects a lack of exposure and knowledge around the issue. When asked for an opinion on whether the trade should be continued, many answered that it depends on other factors. Answering that it “depends” may indicate a deeper level of awareness that it is a complex issue, and a potential acknowledgement of a lack of more complete and balanced information. Although half of respondents that supported an end to the trade were in favour of instant cessation, the strong presence of farmer, economic and job support from those supporting continuation of the trade may suggest that a government concession to a well-managed phase-out of the industry and transition period, in which stakeholders are not negatively affected in the long term, is likely to be more positively received by the general public than an instant ban.

## Figures and Tables

**Table 1:** Description of participant demographics in respondents (*n* = 522) to a survey of public opinion in Brisbane CBD about live export in Australia *.

Demographic Category	Responses	*n*=
Gender	Male	214
	Female	308
Age category	20–29	135
	30–39	141
	40–49	99
	50–59	69
	60+	79
Residential zoning	Rural	75
	Suburban	267
	City	180
Highest education level	Primary School	6
	High School	135
	Technical training college (TAFE)	90
	University degree	168
	Postgraduate	123
Work within livestock industry?	Yes	16
	No	506

* Demographics of Australians in general, according to the Australian Bureau of Statistics, “Census (2016)”.

**Table 2:** Responses to questions about the live export trade in surveys conducted before and after a media event about live export *.

Question	Responses	Total Respondents	Respondents before Media Event	Respondents after Media Event	% Difference	*p* Value (Chi Square)
Are you familiar with the live trade industry?	Yes	394 (74.5%)	201 (71.0%)	193 (78.5%)	+7.4%	0.05 (3.82)
	No	135 (25.5%)	82 (29.0%)	53 (21.5%)	−7.4%	
How do you feel about the live export trade?	Positive	75 (14.3%)	40 (14.2%)	35 (14.3%)	+0.11%	0.16 (3.65)
	Negative	316 (60.2%)	160 (56.9%)	156 (63.9%)	+7.0%	
	No feelings	134 (25.5%)	81 (28.8%)	53 (21.7%)	−7.1%	
Have you seen footage around the live export debate?	Yes	249 (47.4%)	117 (41.8%)	132 (53.9%)	+12.1%	0.006 (7.66)
	No	276 (52.6%)	163 (58.2%)	113 (46.1%)	−12.1%	
Do you believe the Australian live export trade should be ended?	Yes	220 (42.0%)	117 (41.9%)	103 (42.0%)	+0.1%	0.30 (2.43)
	No	97 (18.5%)	58 (20.8%)	39 (15.9%)	−4.9%	
	Depends	207 (39.5%)	104 (37.3%)	103 (42.0%)	+4.8%	
Method of ending the trade	Instantly	112 (50.9%)	55 (47.0%)	57 (55.3%)	+8.3%	
	Phase out	108 (49.09%)	62 (52.99%)	46 (44.66%)	−8.33%	

* Captured only for respondents stating “yes” or “depends” when asked if live export should be ended (*n* = 220).

**Table 3:** The relationship between viewing footage and attitude to live export (Odds Ratio for Yes vs. Depends = 1.51, 95% Confidence Interval 1.03–2.22, *p* = 0.03).

Have You Seen the Footage	“Should Live Export Be Ended”?		
	Yes	Depends	No
Yes	114 (46)	86 (35)	49 (20)
No	106 (39)	121 (44)	48 (17)

**Table 4:** Responses to questions about how respondents * felt about the live export trade in surveys conducted before and after a media event about live export.

Response (in Declining Order of Response Number)	*n*=	%
Sadness	56	22.6%
Empathy for the animals	49	19.8%
Anger	36	14.5%
Sickening	32	12.9%
Disbelief	15	6.0%
Frustration	12	4.8%
Disgusted	4	1.6%
Disappointed	4	1.6%
Irritated at sensationalism	4	1.6%
Shock	3	1.2%
Sick of hearing about it	2	0.8%
Defensive for the live trade industry	2	0.8%
No answer given	2	0.8%
No response	7	2.8%
Free text field, if no suitable response listed	20	8.1%
	**248**	

* Exclusive of those respondents who stated they had *not* recently seen footage surrounding the live export debate (*n* = 282).

**Table 5:** Reasons for answering “no” (*n* = 98) or “depends” (*n* = 205) when asked whether the trade should be ended (Chi-square test of association, Chi-square = 91.7, *p* < 0.001).

Reason	Answered “No”	Answered “Depends”
	No. (%)	No. (%)
Farmer support	44 (44.9)	36 (17.6)
Economy	39 (39.8)	23 (11.2)
Jobs	7 (7.1)	12 (5.9)
Animal-based reasons	4 (4.1)	63 (30.7
Ethical reasons	4	71 (34.6)
**Total**	**98**	**205**

**Table 6:** Probabilities, Odds ratios and 95% confidence intervals for demographic effects on attitudes towards live export, analysed by logistic regression.

Demographic Variable	*p*	Odds Ratio	95% Confidence Interval
***Are You Familiar with the Live Export Trade (Yes, No)?***			
Sex	0.20	1.33	0.86–2.04
Age	<0.001	0.61	0.51–0.72
Have worked in livestock industry	0.13	0.33	0.067–1.62
Rural residential zone	0.36	0.60	0.29–1.26
Education level	0.008	0.38	0.21–0.71
***Feelings (Positive, Negative, Depends) about the Live Export Trade***			
Sex	0.72	0.94	0.66–1.33
Age	<0.001	0.74	0.65–0.84
Have worked in livestock industry	0.04	0.34	0.12–0.97
Rural residential zone	0.05	0.57	0.32–1.01
Education level	0.80	1.06	0.67–1.69
***Opinion on If the Live Export Trade Should Be Ended (Yes, No, Depends)***			
Sex	<0.001	0.43	0.30–0.60
Age	0.17	0.92	0.81–1.04
Have worked in livestock industry	0.10	0.44	0.16–1.17
Rural residential zone	0.08	0.62	0.36–1.05
Education level	0.58	0.88	0.57–1.37
***Have You Seen Recent Footage?***			
Sex	0.70	1.07	0.75–1.54
Age	<0.001	1.32	1.15–1.50
Have worked in livestock industry	0.21	2.00	0.67–5.96
Rural residential zone	0.79	0.89	0.50–1.59
Education level	0.97	0.90	0.56–1.45

**Table 7:** Number of respondents (and per cent) in the different age categories that indicated that they were familiar with the industry, their feeling and whether they did not see the recent footage.

	20–29	30–39	40–49	50–59	60+
Familiar with industry	81 (60)	96 (68)	84 (86)	63 (91)	68 (86)
No feelings	43 (33)	44 (34)	22 (17)	15 (11)	7 (5)
Negative feelings	78 (25)	85 (27)	60 (19)	41 (13)	52 (16)
Positive feelings	12 (16)	12 (16)	17 (23)	13 (18)	20 (27)
Did not see recent footage	82 (30)	84 (31)	51 (19)	27 (10)	30 (11)
